# Angiotensin II Stimulation of DPP4 Activity Regulates Megalin in the Proximal Tubules

**DOI:** 10.3390/ijms17050780

**Published:** 2016-05-20

**Authors:** Annayya Aroor, Marcin Zuberek, Cornel Duta, Alex Meuth, James R. Sowers, Adam Whaley-Connell, Ravi Nistala

**Affiliations:** 1 Divisions of Endocrinology and Nephrology, Department of Medicine, University of Missouri-Columbia, Columbia, MO 65212, USA; aroora@health.missouri.edu (A.A.); cduta@fayettenews.com (C.D.); meutha@health.missouri.edu (A.M.); sowersj@health.missouri.edu (J.R.S.); whaleyconnella@health.missouri.edu (A.W.-C.); 2 School of Medicine, University of Missouri-Columbia, Columbia, MO 65212, USA; mjzg28@health.missouri.edu; 3 Harry S. Truman Memorial Veteran’s Hospital, Columbia, MO 65201, USA

**Keywords:** proteinuria, obesity related kidney disease, megalin, DPP4, ERK, EGFR, AT_1_R

## Abstract

Proteinuria is a marker of incipient kidney injury in many disorders, including obesity. Previously, we demonstrated that megalin, a receptor endocytotic protein in the proximal tubule, is downregulated in obese mice, which was prevented by inhibition of dipeptidyl protease 4 (DPP4). Obesity is thought to be associated with upregulation of intra-renal angiotensin II (Ang II) signaling via the Ang II Type 1 receptor (AT_1_R) and Ang II suppresses megalin expression in proximal tubule cells *in vitro*. Therefore, we tested the hypothesis that Ang II will suppress megalin protein via activation of DPP4. We used Ang II (200 ng/kg/min) infusion in mice and Ang II (10^−8^ M) treatment of T35OK-AT_1_R proximal tubule cells to test our hypothesis. Ang II-infused mouse kidneys displayed increases in DPP4 activity and decreases in megalin. In proximal tubule cells, Ang II stimulated DPP4 activity concurrent with suppression of megalin. MK0626, a DPP4 inhibitor, partially restored megalin expression similar to U0126, a mitogen activated protein kinase (MAPK)/extracellular regulated kinase (ERK) kinase kinase (MEK) 1/2 inhibitor and AG1478, an epidermal growth factor receptor (EGFR) inhibitor. Similarly, Ang II-induced ERK phosphorylation was suppressed with MK0626 and Ang II-induced DPP4 activity was suppressed by U0126. Therefore, our study reveals a cross talk between AT_1_R signaling and DPP4 activation in the regulation of megalin and underscores the significance of targeting DPP4 in the prevention of obesity related kidney injury progression.

## 1. Introduction

Overnutrition and obesity have become a major health problem in the United States as well as worldwide [1,2]. Prevalence rates of obesity in children and adults have increased in the past 2–3 decades, which has been associated with the Western diet (WD), which is high in refined sugars and fat [3]. Concurrently, there has been a rise in the incidence and prevalence of diabetes, hypertension and chronic kidney disease (CKD). Therefore, it is widely thought that the increased prevalence of obesity has contributed to an increased progression of kidney disease to end stage kidney disease and dialysis in the general population.

Inappropriate activation of renin-angiotensin system (RAS) is associated with the cardiovascular pathophysiology in obesity [4,5,6,7,8,9,10]. The RAS has diverse roles beyond its well-known role in the maintenance of blood pressure (BP) and regulation of fluid and salt homeostasis [4,11,12]. For example, Ang II, the main effector protein of the RAS has been shown to induce both glomerular and proximal tubule injury contributing to progression of kidney disease [13,14,15,16]. In this regard, megalin a receptor endocytotic protein expressed in the proximal tubule cells, has been identified as a target protein for Ang II-mediated proximal tubule origins of proteinuria [4,17]. In the proximal tubule, the role of megalin includes the reabsorption of filtered albumin and other low molecular weight proteins and many of its passengers, e.g., albumin, insulin and Ang II, are associated with physiologic regulation of kidney function [4,11,18]. For example, Ang II binds to and is internalized by megalin (18) while signaling through AT_1_R negatively regulates megalin expression at both mRNA and protein levels [17]. Furthermore, Ang II infusion in mice causes megalin and other proteins to co-localize to the low-density microvilli enriched membranes while angiotensin converting enzyme (ACE) inhibition has the opposite effect [19]. Whether change in cellular redistribution has an effect on protein transport is not known.

Ang II infusion also results in redistribution of dipeptidyl peptidase 4 (DPP4) to the low-density enriched membranes of the tubules [19]. DPP4 is a type 2 integral membrane protein that is abundantly expressed on the proximal tubule cell and together with aminopeptidase N, helps in the reabsorption of oligopeptides from the glomerular filtrate into the proximal tubule by virtue of its ectopeptidase activity [20,21]. Furthermore, DPP4 has been shown to regulate the activity of sodium hydrogen exchanger 3 (NHE3), which is also enriched in the low-density enriched membranes in response to Ang II infusion [22]. While it is hypothesized that DPP4 co-localization in the low-density microvilli-enriched membranes may assist DPP4 regulation of NHE3, no such role for DPP4 has been assigned in the regulation of megalin. Rodent models of obesity, exhibit higher kidney and plasma levels of DPP4 and lower levels of megalin in the brush border of the proximal tubule [23,24,25]. In this context, inhibition of DPP4 has been shown to improve kidney injury and proteinuria in obese rodent models [23,24,26,27]. Importantly, human diabetes patients are characterized by elevation in DPP4 in the plasma and urine [28,29]. We have previously observed that DPP4 inhibition is associated with reduction in uric acid, 3-nitrotyrosine levels (marker of oxidative stress) and inflammation in the kidney of obese mice [23]. As Ang II is a known promoter of inflammation, kidney injury and proximal tubule dependent proteinuria, and stimulates the co-localization of megalin and DPP4, we hypothesized that Ang II may regulate both proteins via AT_1_R signaling in the proximal tubule. We further hypothesized that since Ang II downregulates megalin in the proximal tubule cell, and also co-localizes DPP4 and megalin to the low-density enriched membranes, DPP4 activation may regulate megalin protein expression.

Herein, we examined the modulation of DPP4 activity and megalin protein levels in kidney tissue obtained from Ang II-infused mice. We then extended our findings to a opossum proximal tubule (OKP) line with stable expression of rat AT_1A_R. We demonstrate here that Ang II stimulates DPP4 activity likely via AT_1_R-mediated transactivation of epidermal growth factor receptor (EGFR). Furthermore, ERK stimulation may occur both directly via AT_1_R and indirectly via DPP4 activation and signaling. The activation of ERK then leads to reduction in megalin protein levels.

## 2. Results

### 2.1. Kidney-Specific Activation of Renin-Angiotensin System (RAS), Dipeptidyl Peptidase 4 (DPP4) Activity and Suppression of Megalin by Ang II Infusion in Mice

**Figure 1:**
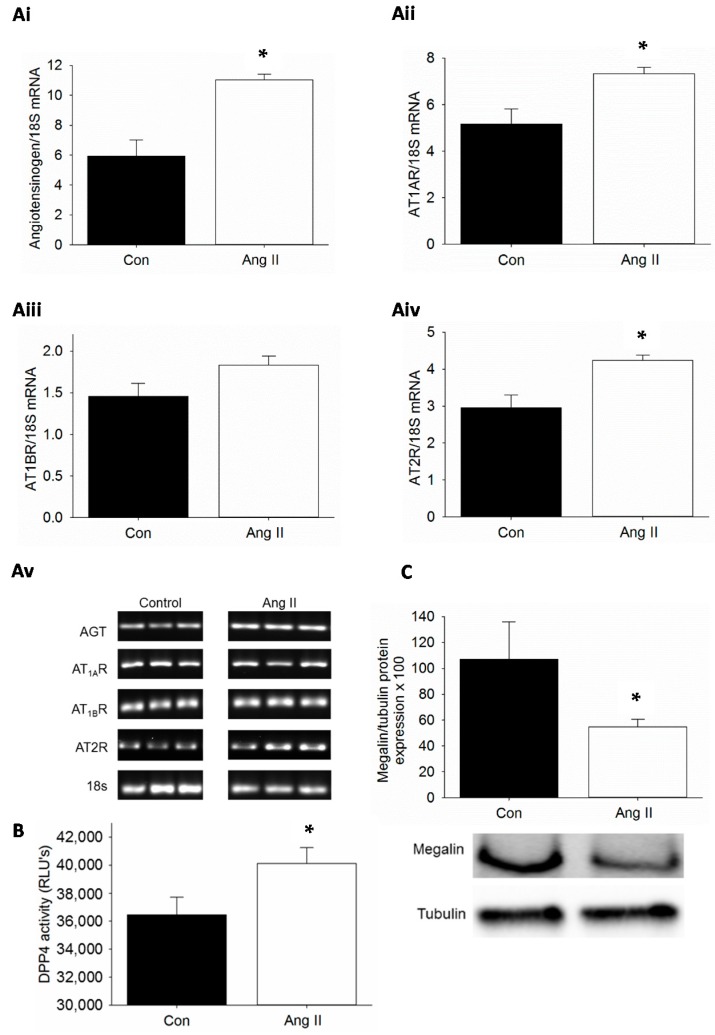
Ang II infusion activates the renin-angiotensin system (RAS) and dipeptidyl peptidase 4 (DPP4) and suppresses megalin protein levels in mice: (**A**) Quantification of differential mRNA expression of RAS in the kidney (**Ai**–**Aiv**) and depiction of actual bands that were used for quantification (**Av**); (**B**) DPP4 activity in the kidney expressed as relative light units (RLUs); and (**C**) megalin protein expression by immunoblot of kidney lysates. *n* = 3–4; * *p* < 0.05; AGT: Angiotensinogen; AT_1A_R: Angiotensin type 1A receptor; AT_1B_R: Angiotensin type 1B receptor; AT2R: Angiotensin type 2 receptor; 18s: 18s ribosomal RNA; Con: Saline-infused mice; Ang II: Ang II-infused mice (200 ng/kg/min).

Obesity is characterized by elevations in plasma Ang II and the thought is that intra-renal RAS is activated as well. In Ang II infusion models, proximal tubule AT_1_R is upregulated as assessed by increased binding of radiolabeled Ang II [13]. Therefore, we examined whether Ang II infusion activated RAS components in the kidney. Low dose Ang II 200 ng/kg/min infusion in C57Bl/6 mice for three weeks did not increase blood pressure as determined by telemetry (data not shown). The mRNA expression of genes for angiotensinogen (*AGT*), *AT_1A_R*, *AT_1B_R* and *AT_2_R* was examined in Ang II-infused mice and compared with saline-infused control mice (Figure 1A). We observed that Ang II infusion resulted in increased expression of *AT_1A_R* with concomitant increase in the expression of AGT gene. There was no significant increase in the expression of *AT_1B_R*. However, Ang II also caused increased expression of *AT_2_R* under conditions of Ang II infusion. We then assessed whether Ang II infusion would stimulate DPP4 activity in the kidney. The effects of non-specific proteases were blocked by the use of protease inhibitors. We observed a significant increase in DPP4 activity in kidney tissue extracts from Ang II-infused mice when compared to saline infused control mice (Figure 1B). Finally, we wanted to test whether Ang II infusion *in vivo* affects megalin protein levels. We have previously reported that megalin protein levels are decreased in proximal tubule cell brush borders of diet induced obesity mice and Zucker obese rat and TG(mRen2) rats [23,24,30]. Others have shown an increase in megalin expression in AT_1A_R knockout mice and with AT_1_R blockade [15,31,32]. Herein, we observed that megalin protein levels were significantly reduced as determined by Western blot analysis in kidney tissue lysates from Ang II-infused mice when compared to saline-infused control mice (Figure 1C).

### 2.2. Proximal Tubule Cell-Specific Increase in DPP4 Activity by Ang II Stimulation

**Figure 2:**
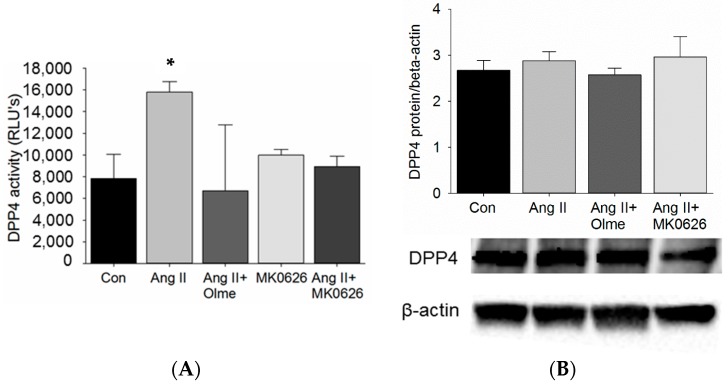
DPP4 enzymatic activity and protein levels in T35OK-AT_1_R proximal tubule cells after acute stimulation with Ang II: (**A**) DPP4 activity after stimulation (30 min) with Ang II (10^−8^ M) and blockade (60 min prior to Ang II) with olmesartan (10^−6^ M) or MK0626 (5 × 10^−6^ M); and (**B**) DPP4 protein levels after stimulation (30 min) with Ang II and blockade with olmesartan and MK0626. *n* = 3–5; * *p* < 0.05; Olme: Olmesartan; MK0626: Rodent DPP4 inhibitor (Merck & Co., Inc.).

Our studies in C57Bl/6 mice showed that Ang II infusion increased DPP4 activity in the kidneys. Furthermore, DPP4 redistributes with megalin to the low-density microvilli-enriched membranes of the proximal tubules in response to Ang II and out of these membranes in response to ACE inhibition. Therefore, we tested whether Ang II stimulation via AT_1_R directly increased DPP4 activity in proximal tubule cells *in vitro*. T35OK-AT_1_R cells with stable AT_1A_R expression were acutely (30 min) stimulated with Ang II (10^−8^ M). The stimulation resulted in an increase in DPP4 activity when compared to untreated controls (Figure 2A) and blockade with an AT_1_R blocker (olmesartan, 10^−6^ M) in another group 1 h prior to the addition of Ang II, reduced DPP4 activity back to baseline. Despite expectation of a much greater inhibition of DPP4 activity by MK0626 (5 × 10^−6^ M) (IC_50_ 6.3 nmol/L), we only observed a reversal back to baseline of increased DPP4 activity in response to Ang II and with no Ang II stimulation (Figure 2A) [23]. In addition, the dependency of increased DPP4 activity on DPP4 protein levels was tested in the same cells and we observed no change in DPP4 protein levels suggesting that the Ang II-mediated increase in DPP4 activity occurs independent of DPP4 protein expression.

### 2.3. Restoration of Megalin Protein Levels by DPP4 Inhibition Is via Suppression of EGFR and ERK Activation in Proximal Tubule Cells

**Figure 3:**
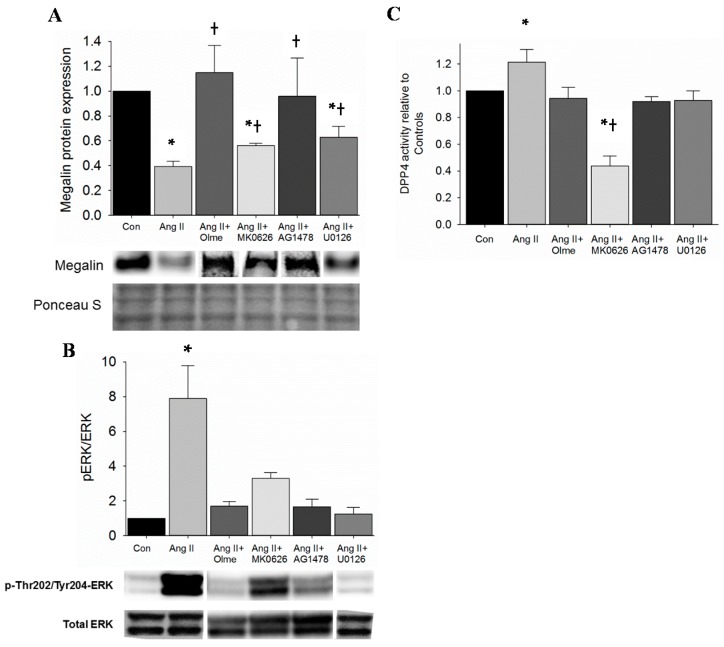
Ang II regulates megalin protein expression via DPP4 activation. (**A**) Megalin protein expression by immunoblot in T35OK-AT_1_R proximal tubule cells. Proximal tubule cells were stimulated with Ang II (10^−8^ M) for 24 h and pre-treated 1 h with olmesartan (10^−6^ M), AG1478 (10^−5^ M), U0126 (10^−5^ M) and MK0626 (5 × 10^−5^ M); (**B**) ERK1/2 activation was assessed by pThr^202^Tyr^204^-ERK1/2 increase. Proximal tubule cells were stimulated with Ang II (10^−8^ M) for 30 min and the ratio of pThr^202^Tyr^204^-ERK1/2 to total ERK1/2 ratio was calculated. Proximal tubule cells were pre-treated for 1 h with olmesartan (10^−6^ M), AG1478 (10^−5^ M), U0126 (10^−5^ M) and MK0626 (5 × 10^−5^ M); (**C**) DPP4 activity in conditions of chronic Ang II stimulation. T35OK-AT_1_R proximal tubule cells were stimulated for 24 h with Ang II (10^−8^ M) and olmesartan (10^−6^ M), AG1478 (10^−5^ M), U0126 (10^−5^ M) and MK0626 (5 × 10^−5^ M) were tested for blockade of Ang II-mediated increase in DPP4 activity. *n* = 3–6; * *p* < 0.05 when compared to control; † *p* < 0.05 when compared to Ang II.

Ang II increased DPP4 activity in the kidney of mice and in proximal tubule cells. In addition, Ang II infusion reduced megalin protein levels in the kidney. Moreover, Ang II-infusion redistributes DPP4 to the low-density microvilli-enriched fractions along with megalin. Therefore, we stimulated T35OK-AT_1_R proximal tubule cells in a chronic manner with Ang II (10^−8^ M) and observed a significant decrease in megalin protein levels as expected (Figure 3A and Figures S1–S8). Furthermore, in support of our hypothesis, megalin protein levels were partially restored by pre-treatment with MK0626 (5 × 10^−5^ M), indicating a potential regulatory link between DPP4 and megalin. In order to better define the mechanism for MK0626-mediated beneficial effect on megalin, we performed the next set of experiments. Classically, Ang II activates extracellular signal-regulated kinase (ERK1/2) through AT_1_R to signal downstream and ERK1/2 has been shown to downregulate megalin expression in proximal tubule cells [17]. Therefore, we examined whether suppression of ERK1/2 activation contributes to DPP4 inhibition-mediated rescue of megalin protein. First, we established that Ang II (10^−8^ M) activates ERK1/2 in T35OK-AT_1_R cells via increase in pThr^202^Tyr^204^-ERK1/2 levels when compared to untreated controls and that U0126 (10 µM), MEK1/2 inhibitor, blocks this activation (Figure 3B); Second, T35OK-AT_1_R cells were treated with Ang II (10^−8^ M) for 30 min and MK0626 (5 × 10^−6^ M) was added 1 h prior. Ang II-mediated ERK1/2 activation as indicated by increase in pThr^202^Tyr^204^-ERK1/2 was partially suppressed by MK0626 treatment of PTCs (Figure 3B); Third, we were also interested to test if ERK1/2 inhibition had any impact on Ang II-stimulated DPP4 activity, which, if present, would show a two way interaction between DPP4 and ERK1/2 proteins. Under control conditions, MEK1/2 inhibitor (U0126) lowered DPP4 activity below baseline (data not shown). In contrast, U0126 lowered Ang II-mediated DPP4 activation back to baseline suggesting that ERK1/2 activation regulates DPP4 activity and also plays a role in Ang II-mediated DPP4 activation; Fourth, we examined the cellular behavior with respect to megalin expression in the presence of U0126 and AG1478, knowing that Ang II can activate ERK1/2 and transactivate the epidermal growth factor receptor (EGFR) (unpublished data). Western blot analysis revealed that U0126 partially restored megalin expression similar to MK0626 (Figure 3A and Figures S1–S8). In comparison, AG148 was able to restore megalin expression to a greater degree than MEK1/2 inhibition alone. Last, we examined the proximal tubule cell responses *in vitro* under chronic Ang II presence to test if continued elevation in DPP4 activity is needed for megalin protein levels to decrease. Compared to untreated controls (Figure 3C), chronic (24 h) Ang II stimulation of proximal tubule cells resulted in an incremental increase in DPP4 activity. In order to better define the mechanisms involved, blockade of AT_1_R, ERK1/2 and EGFR activation was tested. To our surprise, blockade of all three proteins resulted in a decrease in DPP4 activity back to the baseline (Figure 3C). In comparison, under conditions of chronic Ang II stimulation, DPP4 activity was attenuated to a greater extent by MK0626.

## 3. Discussion

In the current study, we demonstrate that DPP4 activation occurs via direct Ang II/AT_1_R signaling *in vitro* and *in vivo*. We further demonstrate that Ang II suppression of megalin protein levels is prevented by DPP4 inhibition and this occurs via suppression of Ang II-stimulated ERK1/2 activation. Moreover, EGFR transactivation by AT_1_R may play a role in the activation of DPP4 and ERK1/2, which in turn may lead to reduction in megalin protein levels.

Our observation that Ang II increases DPP4 in kidney tissue lysates and in cultured proximal cells is novel. To our knowledge, although Ang II crosstalk with insulin in the genesis of insulin resistance has been studied in detail, the role of Ang II in the regulation of DPP4, a potential player in mediating insulin resistance, has not been studied. In this regard, these data demonstrate that DPP4 is regulated in response to insulin and TNF-α (released from differentiated adipocytes) [33]. In addition, when recombinant DPP4 is added to adipocytes, it disrupts insulin-mediated phosphorylation of Akt suggesting that overexpression of DPP4 in conditions of obesity and diabetes may result in insulin resistance. Insulin infusion has been shown to tyrosine phosphorylate DPP4 via association with c-Src in the Golgi/endoplasmic reticulum of liver tissue, suggesting that there is a pool of Src kinase that is amenable to stimulation by peptide agonists [34]. Moreover, Src kinase has been shown to have a major role in orchestrating tyrosine phosphorylation of its target proteins (EGFR and Cav-1) in caveolae and is a downstream effector of Ang II signaling cascades. In this study, we observed downregulation of DPP4 activity in response to AG1478, a potent EGFR inhibitor and Ang II transactivation inhibitor, which suggests that suppression of DPP4 phosphorylation via either EGFR-mediated cascades or Src tyrosine kinase may regulate DPP4 activity. The classical EGFR-mediated activation of ERK was tested in this study since ERK phosphorylation was shown to be associated with reduction in megalin [17]. Other conditions that could potentially activate DPP4 are hypoxia and/or oxidative stress. In the case of hypoxia, the results have been conflicting so far with one study showing no effect in adipocytes and another showing activation in DPP4. In addition to agonists regulating DPP4 activity, what the agonists do to DPP4 mRNA and/or protein is of tremendous interest [35,36]. Active DPP4 is a homodimeric structure, with monomeric DPP4 having only residual activity. Dimer formation requires hydrophobic interactions [37] and the interaction between dimers is critical for a successful enzymatic activity. It has been shown that conformational changes with impact on activity are propagated from the transmembrane domain to the extracellular active site [38]. In addition, DPP4 activity is regulated through glycosylation in diabetic nephropathy [39]. In the case of Ang II, this modulation could be exerted either directly or through an intermediate molecule, part of the cell-signaling cascade through AT_1_R.

We observed that Ang II infusion into mice resulted in activation of kidney RAS. It is known that most Ang II effects are mediated through binding to its specific receptors (AGTRs), and of these, AT_1_R, a subset of AGTR, are thought to be responsible for the pro-growth, hypertrophy, inflammation, vasoconstriction, thirst and sodium retaining effects of Ang II. In addition, Ang II may exert a positive feedback on various RAS components, leading to a further increase in its local concentrations and that of AGT and AT_1A_R (sub-type of AT_1_R) has been observed by others [40,41], and was confirmed in our present study (Figure 1). Since AT_2_R effects are generally considered to antagonize the effects of AT_1_R, their expression should follow a similar trend; that is, if AT_1_R are up-regulated, the number of AT_2_R will also increase to counterbalance AT_1_R. In our kidney extract samples, we found increases in the expression of both *AT_1A_R* and *AT_2_R*, thus confirming in part our expectations, although *AT_1B_R*, another subset of AT_1_R, did not increase significantly upon low dose Ang II stimulation. AT_1_R sub-types are functionally similar and not distinguishable pharmacologically, however our observation suggests they may be regulated differently [42]. The line of evidence about AT_1_R sub-types and their expression is limited, and, although our present work does not explore the consequences of differential expression of AT_1_R, these findings are interesting.

The specific sequence of intracellular signaling events that follow AT_1_R activation in specific cell types and conditions is still an area of active research. Experimental findings in the literature suggest that transactivation of EGFR by Ang II plays an important role in ERK1/2 activation and activation of ERK1/2 by Ang II has been shown to modulate proximal tubule function including megalin expression [17,43]. In our study, the addition of an EGFR inhibitor (AG1478) prior to Ang II, prevented an increase in DPP4 activity beyond baseline (Figure 3C), an effect similar to AT_1_R blockade through olmesartan, supporting a prominent role for EGFR transactivation in Ang II-activation of DPP4. In addition, AG1478 prevented ERK1/2 phosphorylation and decrease in megalin protein levels, further implicating EGFR transactivation by the AT_1_R as a major player.

The role of megalin in the reabsorption of various molecules at the proximal tubule brush border such as albumin and other low molecular weight proteins is well known. In contrast, the regulation of megalin in conditions of obesity and RAS activation is not well known. In one study, Ang II downregulated megalin through ERK1/2 activation and insulin/PI3-K antagonized Ang II-mediated effects and preserved megalin protein levels, partially [17]. In that study, blockade of Ang II-AT_1_R downstream targets including c-jun N-terminal kinase (JNK), protein kinase C (PKC), Janus kinase (JAK) and p38 mitogen activated protein kinase (p38MAPK) did not restore megalin levels. However, it is not known how EGFR and DPP4 activation regulate megalin. Ang II activation of EGFR plays an important role in proximal tubule pathology and albuminuria through activation of TACE (tumor necrosis factor-α converting enzyme, also known as ADAM17) [44]. We have observed that Ang II-mediated EGFR transactivation and ERK activation, may regulate albumin endocytosis via NADPH oxidase-mediated production of reactive oxygen species (manuscript in preparation). Furthermore, reactive oxygen species production via NADPH oxidase may regulate megalin. However, the role of DPP4 and its enzymatic activity in megalin regulation was not addressed in that study. Herein, we show that Ang II-EGFR increase in DPP4 activity leads to ERK phosphorylation and that reduction in ERK phosphorylation inhibits increases in DPP4 activity suggesting a 2-way communication between these pathways. Increase in DPP4 enzymatic activity is thought to be associated with increased function of DPP4 and in this case DPP4 signaling may be enhanced via Ang II-EGFR pathway. DPP4 signaling has been shown to decrease phosphorylation of Akt and lead to insulin resistance in adipocytes [33]. However, we have not addressed the downstream effects of recombinant DPP4 in our study. Interestingly, we observed that MEK inhibitor U0126, was able to suppress Ang II-mediated increase in DPP4 activity. We think that this suppression of DPP4 activity by U0126 could be due to non-specific inhibition either through enhanced degradation of DPP4 or reduced phosphatases or increased shedding. Very little is known regarding the co-localization of megalin and DPP4 in the proximal tubules and this association is probably not fortuitous since many of the Ang II-AT_1_R downstream signaling molecules such as PKC, PI3-K, mTOR and ERK1/2 regulate the expression and functions of megalin [17,19,45].

Based on our results, we suggest a new Ang II signaling model to DPP4 and megalin. Ang II, increased in states of chronic RAAS activation, exerts its effects through AT_1_R, which once activated, transactivates EGFR and leads to phosphorylation of ERK. The activation of DPP4 by Ang II occurs through EGFR and ERK1/2 activation. DPP4 also modulates ERK1/2 and, DPP4-mediated reduction of megalin protein partially occurs though ERK1/2 dependent mechanisms. Further work needs to be done to characterize whether *in vivo* treatment with MK0626 can reverse Ang II-mediated increases in kidney DPP4 activity and reductions in megalin protein. In summary, targeting DPP4 may be a novel way of suppressing renal injury under the setting of inappropriate renal RAS activation such as obesity and diabetes.

## 4. Experimental Section

### 4.1. Animals

Male C57Bl/6 mice were purchased from Charles River, Inc. (Wilmington, MA, USA) and cared for in accordance with National Institutes of Health guidelines. All procedures were approved by the Institutional Animal Care and Use Committee of the University of Missouri. Mice were divided into two groups to include C57Bl/6 control (Con) and C57Bl/6 treated with Ang II 200 ng/min/kg (Ang II).

### 4.2. Proximal Tubule Cell Culture

T35OK-AT_1_R proximal tubule cells (kind gift from Thomas Thekkumkara, TTUHSC School of Pharmacy, Amarillo, TX, USA) stably expressing rat AT_1A_R were grown in DMEM/F12 with 10% fetal bovine serum (FBS), insulin, transferrin, dexamethasone, EGF and G418 to maintain selection pressure. Proximal tubule cells grown in 100 mm dishes, were starved overnight in DMEM/F12 with 0.1% FBS and treated with Ang II (10^−8^ M) for 24 h. Various inhibitor treatments were performed 1 h prior to adding Ang II where indicated. AG1478 and olmesartan was obtained from Sigma (St. Louis, MO, USA). U0126, was procured from Tocris (Bristol, UK).

### 4.3. Preparation of Kidney Tissue and Cell Extracts

Frozen kidney tissue and proximal tubule cell extracts were homogenized in ice-cold modified RIPA buffer containing 1% triton X-100, 100 mM NaCl, 20 mM Tris pH 7.5, 2.0 mM EDTA, 10 mM MgCl_2_, 10 mM NaF, 40 mM β-glycerol phosphate, 1 mM PMSF, 2 mM sodium orthovanadate, protease inhibitor cocktail tablet (Roche Diagnostics, Indianapolis, IN, USA), 10 µg/mL leupeptin, 7 µg/mL pepstatin. Homogenates were centrifuged at 13,200× *g* for 10 min and the supernatant collected. Protein concentrations were determined using a BCA protein assay kit (Thermo Scientific, Rockford, IL, USA).

### 4.4. Immunoblotting

Forty micrograms of proteins were separated by SDS-PAGE and transferred to nitrocellulose membranes and blocked for 1 h at RT according to manufacturer’s instructions. For megalin, NuPAGE large protein gel separation kit was used and manufacturer’s instructions (Invitrogen, Carlsbad, CA, USA) were followed to prepare samples. Protein transfer to nitrocellulose was done overnight at 15 V at 4 °C. Primary antibodies for pERK1/2, ERK1/2 were from Cell Signaling (Danvers, MA, USA), anti-rat megalin antibody was from Santa Cruz Biotechnology (Dallas, TX, USA) and DPP4 antibody was from Origene Technologies, Inc. (Rockville, MD, USA). Anti-opossum megalin antibody was a kind gift from Daniel Biemesderfer (Yale University, New Haven, CT, USA). Antibody binding was detected by chemiluminescence and images recorded using a Bio-Rad ChemiDoc XRS image analysis system (Bio-Rad Laboratories, Santa Cruz, CA, USA). Protein band density quantitation was performed using Image Lab software (Bio-Rad Laboratories).

### 4.5. DPP4 Activity Assay

Kidney tissue [23] and T35OK-AT_1A_R cell extracts were prepared as previously described and as above, respectively. Kidney tissue extracts, prepared as before, were thawed on ice. For the activity assay, 100 µg protein was resuspended in DPP4 assay buffer (Tris-HCl pH 8.0, NaCl 150 mM and protease inhibitor cocktail (Roche Diagnostics, Indianapolis, IN, USA)) in a black 96-well plate for a final volume of 50 µL. Fifty microliters of substrate, 200 mM H-Ala-Pro-AFC (I-1680, Bachem, Torrance, CA, USA), was added to each well and the plate incubated for 10 min at room temperature in the dark. Appropriate positive and negative controls were set up with and without DPP4 inhibitor. Fluorescence was measured with a Synergy Microplate Reader (Biotek, Winooski, VT, USA) at excitation wavelength of 405 nm and emission wavelength of 535 nm. Results are reported as relative light units (RLUs).

### 4.6. Semi-Quantitative Polymerase Chain Reaction

Total RNA from kidney tissue was extracted using the TRIzol reagent (Invitrogen) followed by purification on RNeasy midi columns (Qiagen, Valencia, CA, USA) including DNAse treatment. cDNA was synthesized from 2 µg of total RNA using the ImProm-II cDNA synthesis kit (Promega, Madison, WI, USA). Reverse transcriptase-polymerase chain reaction (RT-PCR) reaction was performed using Platinum Blue PCR mix (Invitrogen) as per manufacturer’s instructions. The primers used for RT-PCR are based on published sequences with the following modifications [46]. Thermal cycling conditions were 95 °C for 5 min (hot start), followed by 35 cycles of 95 °C for 15 s denaturation, and 57 °C for 1 min annealing and extension. The PCR products were electrophoresed on 1.5% agarose gel and bands visualized using Biorad Imaging System. The intensity of the bands were quantitated by densitometric readings in Imagelab. The expression of each gene was normalized to 18S RNA and calculated relative to control animal.

### 4.7. Statistical Analysis

Results are reported as the mean ± SEM. One way ANOVA and *post hoc* *t*-tests (Fisher’s LSD) were performed to examine differences in outcomes between different groups. Unpaired *t*-tests were used where indicated. *p* values of <0.05 are considered significant.

## 5. Conclusions

Ang II stimulates DPP4 activity in the proximal tubules via activation of EGFR and/or ERK pathways likely independent of a change in DPP4 protein expression. DPP4 inhibition partly suppresses ERK activation, which restores Ang II reduction in megalin protein levels. DPP4 may also mediate ERK independent reduction in megalin protein levels. Taken together, DPP4 plays an important modulatory role in megalin protein regulation, which underscores the utility of DPP4 inhibition in the management of kidney injury/proteinuria in conditions of RAS activation.

## Supplementary Materials

Supplementary materials can be found at http://www.mdpi.com/1422-0067/17/5/780/s1.
