# Supplementary material for: Angiotensin II Stimulation of DPP4 Activity Regulates Megalin in the Proximal Tubules
Source: Int J Mol Sci. 2016 May 20;17(5):780. doi: 10.3390/ijms17050780 (PMC4881597; doi:10.3390/ijms17050780)
Supplement: Supplementary file 1 [file ijms-17-00780-s001.pdf]

Supplementary Materials: Angiotensin II Stimulation of DPP4 Activity Regulates Megalin in the Proximal Tubules

Annayya Aroor, Marcin Zuberek, Cornel Duta, Alex Meuth, James R. Sowers, Adam Whaley-Connell and Ravi Nistala

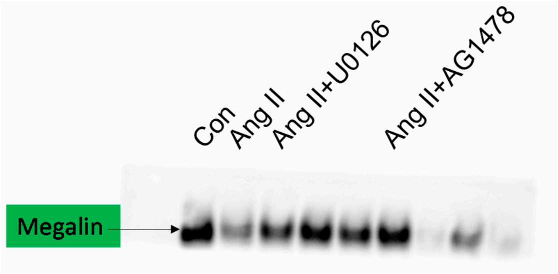

Figure S1. Source file for Megalin band in Con, Ang II & Ang II+U0126 samples–Western blot.

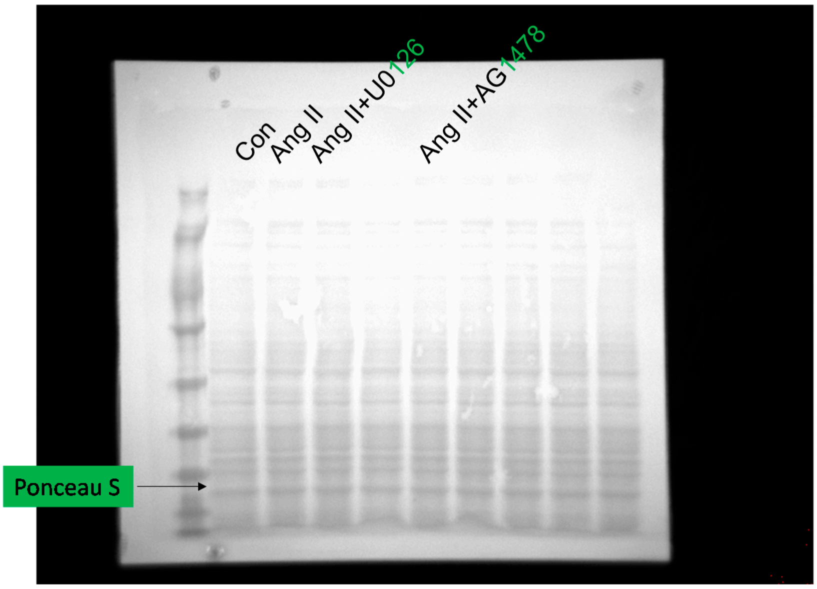

Figure S2. Source file for Ponceau S bands in Con, Ang II & Ang II+U0126 samples–Western blot.

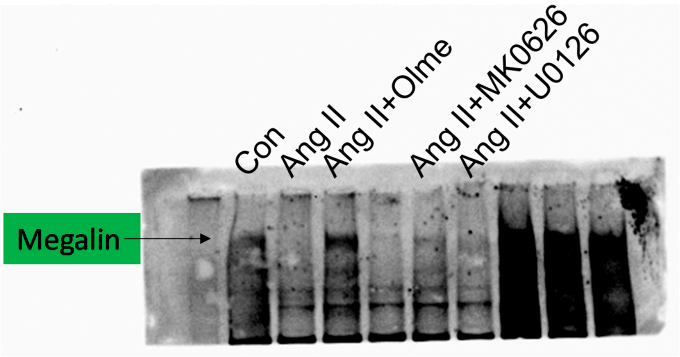

Figure S3. Source file for Megalin band in Ang II+Olme sample–Western blot.

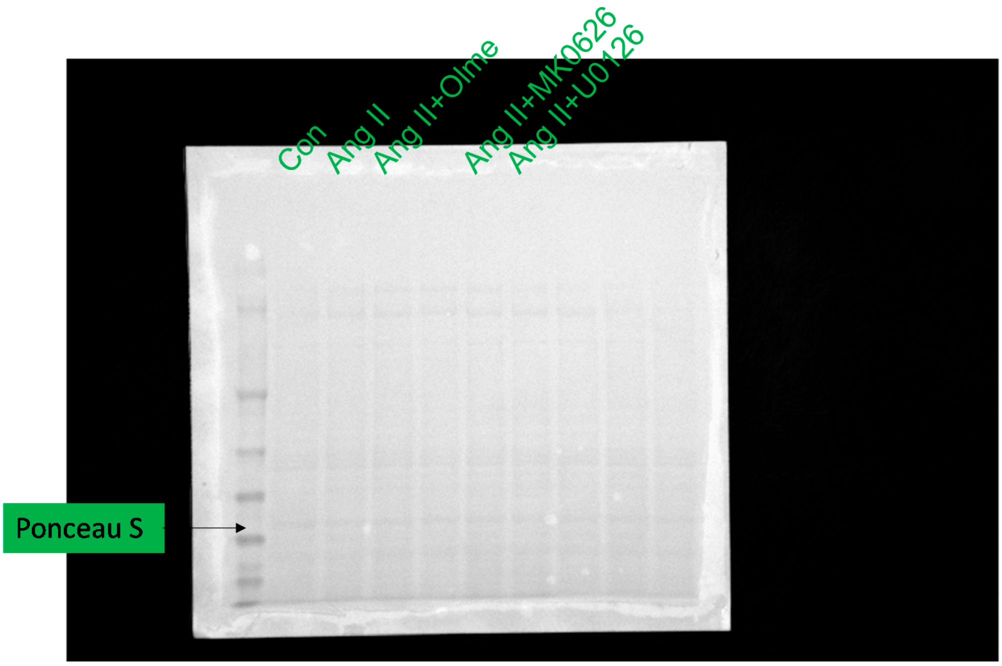

Figure S4. Source file for Ponceau S bands in Ang II+Olme sample–Western blot.

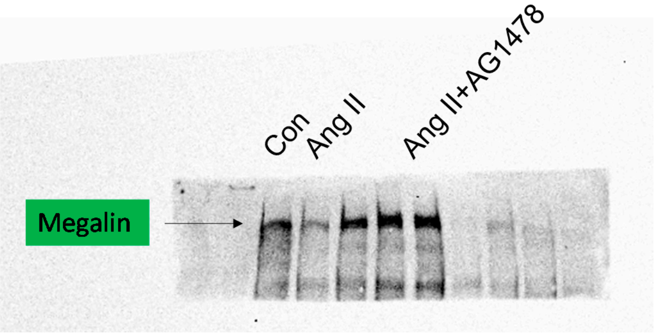

Figure S5. Source file for Megalin bands in Ang II+AG1478 sample–Western blot.

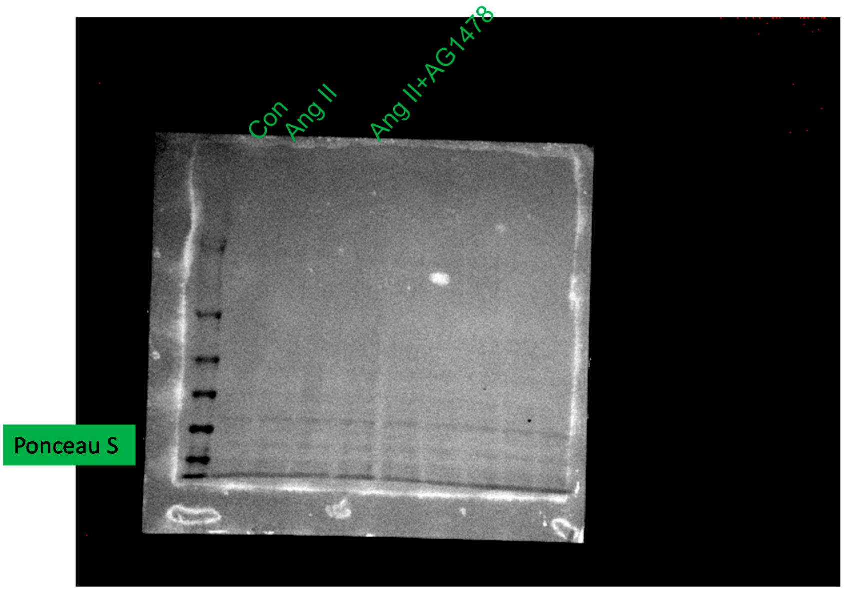

Figure S6. Source file for Ponceau S bands in Ang II+AG1478 sample–Western blot.

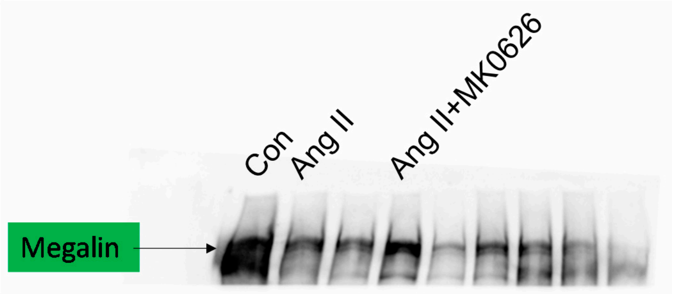

Figure S7. Source file for Megalin bands in Ang II+MK0626 sample–Western blot.

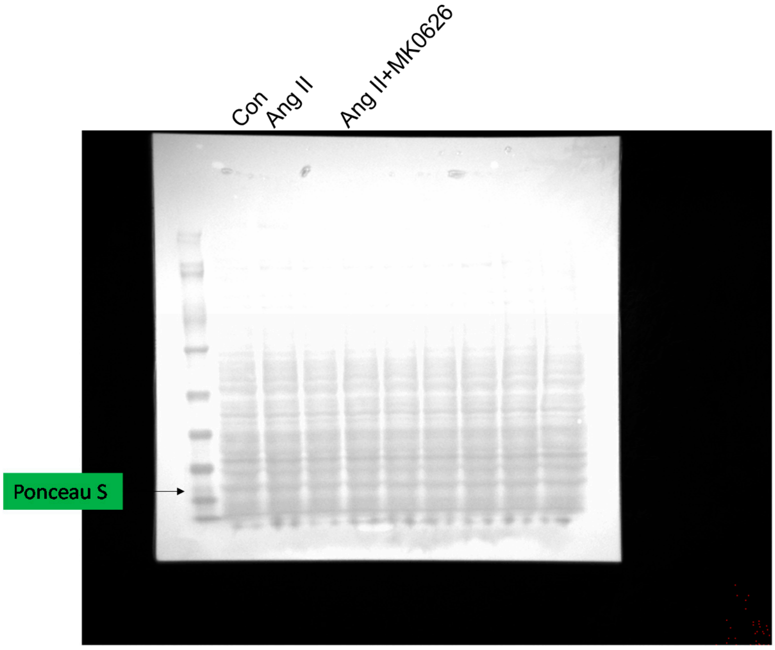

Figure S8. Source file for Ponceau S bands in Ang II+MK0626 sample–Western Blot.
